# Path Planning Generator with Metadata through a Domain Change by GAN between Physical and Virtual Environments

**DOI:** 10.3390/s21227667

**Published:** 2021-11-18

**Authors:** Javier Maldonado-Romo, Mario Aldape-Pérez, Alejandro Rodríguez-Molina

**Affiliations:** 1 Postgraduate Department, Instituto Politécnico Nacional, CIDETEC, Mexico City 07700, Mexico; maldape@ipn.mx; 2 Tecnológico Nacional de México/IT de Tlalnepantla, Research and Postgraduate Division, Estado de México 54070, Mexico; alejandro.rm@tlalnepantla.tecnm.mx

**Keywords:** autonomous driving, machine learning, computer vision, virtual training

## Abstract

Increasingly, robotic systems require a level of perception of the scenario to interact in real-time, but they also require specialized equipment such as sensors to reach high performance standards adequately. Therefore, it is essential to explore alternatives to reduce the costs for these systems. For example, a common problem attempted by intelligent robotic systems is path planning. This problem contains different subsystems such as perception, location, control, and planning, and demands a quick response time. Consequently, the design of the solutions is limited and requires specialized elements, increasing the cost and time development. Secondly, virtual reality is employed to train and evaluate algorithms, generating virtual data. For this reason, the virtual dataset can be connected with the authentic world through Generative Adversarial Networks (GANs), reducing time development and employing limited samples of the physical world. To describe the performance, metadata information details the properties of the agents in an environment. The metadata approach is tested with an augmented reality system and a micro aerial vehicle (MAV), where both systems are executed in an authentic environment and implemented in embedded devices. This development helps to guide alternatives to reduce resources and costs, but external factors limit these implementations, such as the illumination variation, because the system depends on only a conventional camera.

## 1. Introduction

Robotic systems were instrumental in developing tasks that require precision, decreased time, high demand, and cost reduction. According to [1], a robotic system is a reprogrammable, multifunctional manipulator that is designed to move materials, parts, tools, or specialized devices through variable programmed movements which perform different tasks. Besides, manufacturing, agriculture, mining, exploration, and transportation fields implement robotic systems for employing their activities. Therefore, these kinds of systems require specialized equipment [2].

Although different fields implement robotic systems, the systems are high in cost and development. Nevertheless, a robotic system can get advanced features based on the definition of artificial intelligence (AI): it is the field of science that helps machines in the ability to improve their functions; in areas of logic, reasoning, planning, learning, and perception [3]. Hence, the development of robotic systems that interact with the environment uses specialized sensors, localization algorithms, collision detection, and planning of the tasks to be performed [4].

**Figure 1:**
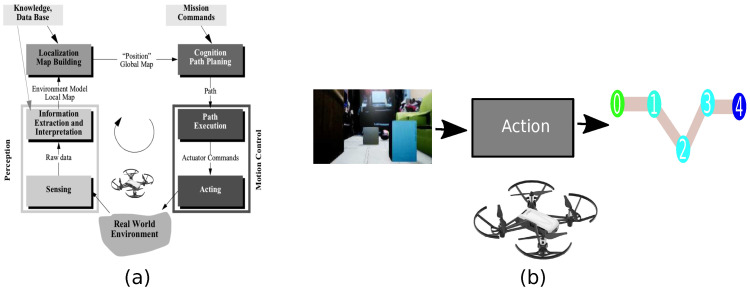
Path planning architecture. (**a**) Standard architecture. (**b**) Reduced architecture.

One of the elementary issues of autonomous robotic systems is the path planning problem. This problem consists of the perception of an environment to generate a path that avoids obstacles. Likewise, it has mainly two modules based on the definition of AI. The first is the planner, while the second module is the perception of the environment, as shown in Figure 1a [5]. The problem is complex according to the robotic system’s characteristics, such as the battery, dimensions, and technologies needed to tackle the perception of the environment, including obstacles, illumination, and other agents in motion.

Due to the high requirements in complex systems, the end-to-end approach has an essential role because this approach consists of reducing external elements such as sensors [6]. Moreover, another approach to minimize the resources is the transfer learning approach. Transfer learning consists in replacing a principal solution with a solution with fewer features [7]. Therefore, a complex architecture can be replaced by a novel architecture that includes the sensors and minimizes the runtime, as shown in Figure 1b.

On the other hand, virtual reality (VR) was intensely used to simulate alternative solutions, which is a kind of technology that enables users to obtain a sensory experience on real things in a similar way to the one that they are in a physical world [8]. Hence, it is possible to represent a virtual representation from an authentic environment for designing, training, improving physical elements. For example, this development uses a virtual environment to train agents [9]. Another technology that enriches experience is augmented reality (AR), which combines the real and virtual, adding extra information using virtual items. In this way, it exists in a real-time interaction, generating an enrichment in user experiences with real scenarios [10].

In previous work [11], a coefficient was introduced to define a limited number of samples of the physical world to estimate a path in a 2D plane. Likewise, an analysis determined that a path generator based on autoencoder estimates the best path compared to that of other approaches.

As a consequence of the above, this proposal employs computer vision techniques for connecting an authentic environment with a virtual representation that associates the path in a 3D environment by a GAN (Generative Adversarial Network) [12]. The interoperability coefficient is used to determine a minimum number of samples in a 3D space. Likewise, we propose metadata to add more information to discrete algorithms, such as the $A^{*}$ algorithm to indicate through colors features that describe the actions of an agent moving in a scenario, such as speed, time, and distribution of objects, to build and offer augmented experiences, since the metadata would provide information to know the composition of the path visually.

This research is composed of the following contributions. A path planer generator with metadata information is introduced. Furthermore, a minimum number of physical samples is determined for connecting with its virtual representation using the interoperability coefficient in 3D space. Subsequently, an augmented reality experience is employed using a mobile device and an embedded application to control a physical micro aerial vehicle (MAV) to evaluate the performance in an authentic environment at runtime.

The remainder of the manuscript is organized as follows. The background and research gaps are described in Section 2. Likewise, the proposed work is introduced in Section 3.2 and Section 4. Subsequently, Section 5 describes the experimental results and analysis. Finally, the conclusions are presented in Section 6.

## 2. Background and Research Gaps

**Figure 2:**
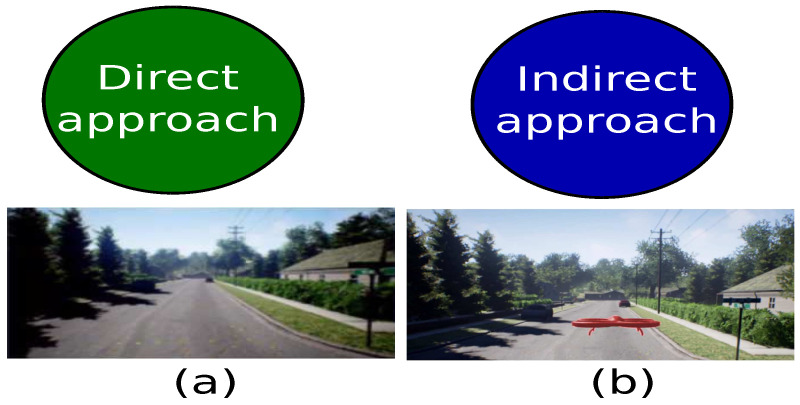
Path planning approaches. (**a**) Direct approach. (**b**) Indirect approach.

According to [13], the path planning problem can be analyzed through two approaches: the first one, called direct form, considers the robotic system as a point of reference, while the second, called indirect form, focuses on the navigation environment as shown in Figure 2.

In the direct approach, the kinematic movements of the robotic system describe the form to interact with the environment. Some of the solutions with a direct perspective approach are dynamic programming [14], soft robotics designing [15] and differential systems [16]. On the other hand, the indirect perspective considered the environment as the principal element to define the agent’s movements. Hence, the environment’s sensing is a high priority. The indirect approach contributions are sampling-based algorithms [17], node-based algorithms [18], and bio-inspired algorithms [19].

The algorithm $A^{*}$ is used to find short paths using graphs. A graph is a set of nodes connected between them. Each node is a tuple that indicates the destination node and the weight that describes the connection. One of the particular algorithm’s characteristics is the movement, highlighting the diagonal between two points. The different movements are based on 45 degrees, forming a star. The heuristics define the complexity of the $A^{*}$ algorithm having a polynomial-time with the implementation of the following expression (1) [20].

(1) $$\left|h\left(x\right)-h^{\prime }\left(x\right)\right|=O\left(log\left(h^{\prime }\left(x\right)\right)\right)$$

Likewise, computer vision processing was used to analyze and find features on input data [21,22,23,24]. Image analysis was studied and generated specialized datasets to perceive the environment, such as NYU Depth Dataset V2 [25], KITTI [26], and Cityscapes [27]. However, these datasets require interaction with real environments. Therefore, the systems require specialized elements to scan information as RGB-D, lidar, or radar sensors.

In the same way, machine learning (ML) algorithms were widely used in classification [28,29] and regression tasks [30,31]. As a result of the intersection of both paradigms, another research area, called generative modeling, emerged. Generative modeling uses Generative Adversarial Networks (GANs) to generate realistic examples across various problem domains. This kind of network automatically learns the regularities in input data so that the model can be used to generate new examples that could plausibly be drawn from the original dataset.

**Figure 3:**
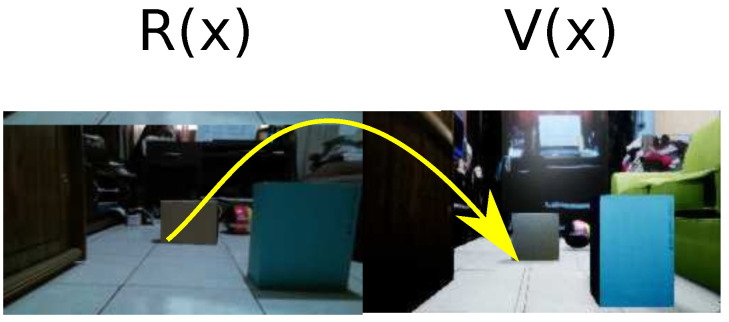
GAN’s domain change from a real environment to its virtual representation.

GANs are based on a competition approach between two types of neuronal networks: generative and discriminative network [32]. The first one is responsible for generating data from a noisy source while the discriminative network is in charge of extracting a set of known characteristics of examples to validate the generator model [33]. The generative network creates candidates, while the discriminative network evaluates them. Besides, GANs were also used for image transformation to map data into a different domain [34] and generate data to create an image with different machine learning approaches [35,36], as shown Figure 3.

### GAN Cost Functions

The system requires an adequate implementation of a GAN that allows generating a change of a domain to another. GANs contain regularization terms that allow for adequate training. This set of rules is called the cost function. To get better performance, an optimization process needs to be incorporated. Specifically, this process involves the maximization of the generator network cost function $G_{1,2}$, the minimization of the discriminator cost function $D_{1,2}$, and the minimization of the noise source cost function $Z_{1,2}$. The cost functions of a GAN network are derived from the calculus of entropy [37]. Thus, the three cost functions are defined as follows [38].

**Definition** **1.** *Let n be the number of samples, let $D_{1,2}$ be the cost function of the discriminator network, let $G_{1,2}$ be the cost function of the generator network, and let $Z_{1,2}$ be the noise source. Maximization of the cost function of the discriminator network is obtained according to the following expression:*

(2) $$M_{cf_{D_{1,2}}}=\frac{1}{n}\cdot \sum_{i=0}^{n}log\left(D_{1,2}\left(i\right)\right)+log\left(1-D_{1,2}\left(G_{1,2}\left(Z_{1,2}^{i}\right)\right)\right)$$

**Definition** **2.** *Let n be the number of samples, let $D_{1,2}$ be the cost function of the discriminator network, let $G_{1,2}$ be the cost function of the generator network, and let $Z_{1,2}$ be the noise source. Minimization of the cost function of the generator network is obtained according to the following expression:*

(3) $$m_{cf_{G_{1,2}}}=\frac{1}{n}\cdot \sum_{i=0}^{n}-log\left(D_{1,2}\left(G_{1,2}\left(Z^{i}\right)\right)\right)$$

**Definition** **3.** *Let $D_{1,2}$ be the cost function of the discriminator network, let $G_{1,2}$ be the cost function of the generator network, and let $Z_{1,2}$ be the noise source. The full cost function of a simple GAN architecture is obtained according to the following expression:*

(4) $$GAN_{cf_{1,2}}=M_{cf_{D_{1,2}}}+m_{cf_{G_{1,2}}}$$

Although this contribution implements GANs to reduce resources [39], the system is employed in physical environments. Therefore, it requires specialized elements to interact with authentic environments.

The following contributions propose the mix of virtual with the real world. However, these works were designed for persons interact with the environment: inverse augmented reality [40], dual reality [41], and one reality [42]. Therefore, their scope is limited for autonomous agents.

In summary, the previous contributions have adequate performance for ground mobile robotics and person interactions because their designs can support more than the limited features of the MAVs, such as flight time of approximately 15 min, small dimensions, and sensors as a conventional camera and an IMU composed by a gyroscope and an accelerometer. For this reason, the analysis of the environment has vital importance in executing an action. However, lidar, RGB-D, infrared, ultrasonic, radars, and 4D radars require specific processing time, much energy, and big dimensions. Consequently, this solution is nonviable for MAVs.

The detected gap in the previous works is the use of specialized elements and high performance. Likewise, the previous works use a physical environment, but virtual simulators can help reduce processing, time, and costs. Therefore, this experiment pretends to use conventional robotic systems to enhance their characteristics, improving them with novel features with metadata. Moreover, a virtual reality simulator with the framework AirSim [43] has an acceptable behavior to simulate a quad-rotor for generating the dataset. On the other hand, GANs have an essential role in changing the domain between the real sample and virtual representation because in a virtual domain are generated whole the possible paths determined by $A^{*}$ algorithm. Additionally, an interoperability coefficient determines the minimum number of samples of an authentic environment. Ultimately, the development is tested in an physical environment using an augmented reality system and a MAV to measure the path planning problem.

## 3. Proposed Work

This section describes the path planner generator with metadata and the generation of the dataset. Moreover, the architecture employed by the *end-to-end* and *transfer learning* approaches is defined.

### 3.1. Domain Connection by GAN Approach

Despite using virtual simulators to design and train algorithms, the analysis performed by the agents in the virtual world is implemented in a real environment again, making the analysis twice. For this reason, it is proposed to make a connection between the real world with the generation of a dataset in the virtual world, which we called *domain connection by GAN* (Equation (5)) where a real sample has an associated virtual representation.

(5) D:R→V

**Figure 4:**
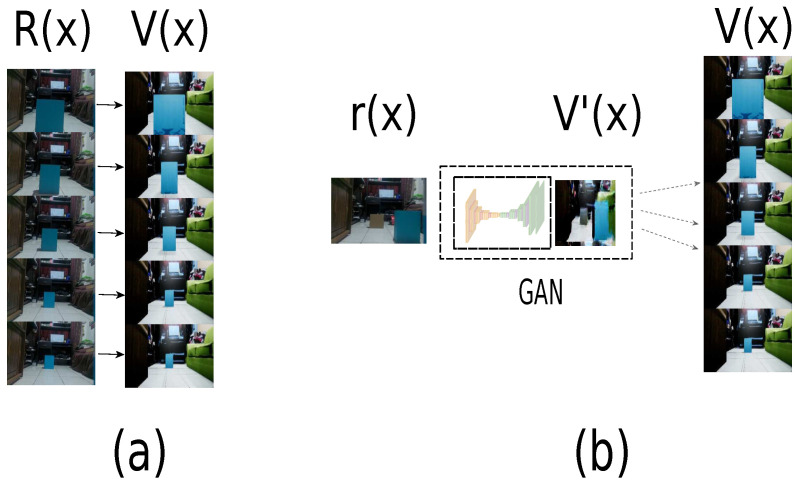
Domain connection approach. (**a**) Connection one-to-one. (**b**) Connected domains by a GAN.

Domain *R* defines the real-world samples, and domain *V* defines the virtual world samples. Therefore, a connection between a real-world sample and the virtual world must generate a one-to-one connection, as is shown in Figure 4a. In other words, this connection must have the same number of samples in both domains, being impractical. On the other hand, GAN is used for a domain change. Therefore, it is possible to change domain from a subdomain *r* with limited samples to virtual domain V (Equation (6)), reducing the samples of domain *R* shown in Figure 4b.

(6) D:r→V

To test the performance of this approach, the path planning problem is implemented using metadata to provide more information to the path, end-to-end, and transfer learning techniques to implement the solution in embedded systems. This experiment is limited to a known and controlled environment.

### 3.2. Path Planner Generator with Metadata

An agent succeeded in different problems of displacement between two points, minimizing resources and physical elements. For example, in the field of robotics, this issue appears from arms [44] to mobile systems [45]. Likewise, the calculus of variations defines the displacement (Equation (7)) between two points as the sum of the distances of two consecutive points, as Equation (8) [46]. Consequently, a path is defined as a set of lines in a space. However, the path planning problem depends on the situation and requirements, such as the agent’s characteristics and the environment.

One of the principal features to describe a path is the level of safety for avoiding obstacles. Therefore, path planning is the shortest distance between the *m* number of obstacles *O* and the best value with the high level of safety in a sequence of points *p* of length *n*. After adding a negative sign to the value, the maximum optimization safety problem is transformed into the minimum optimization problem defined in Equation (9) [47].

(7) $$distance\left(p_{i},p_{i+1}\right)=\sqrt{{\left(x_{i}-x_{i+1}\right)}^{2}+{\left(y_{i}-y_{i+1}\right)}^{2}}$$

(8) $$length\left(p\right)=\sum_{i=0}^{n}distance\left(p_{i},p_{i+1}\right)$$

(9) $$safety\left(p\right)=-\min\min\left\{minDistance(p_{i}p_{i+1},O_{j})\right\}$$

**Figure 5:**
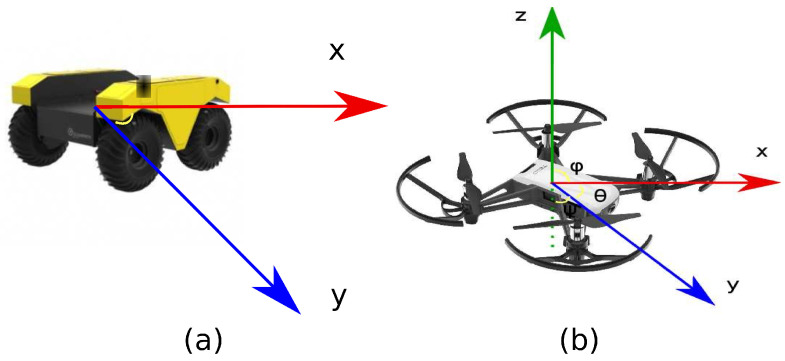
Kind of agents based on movements. (**a**) 2D agent. (**b**) 3D agent.

The complexity of the problems increases based on the number of agents’ movements. For example, Figure 5a shows the movement’s directions on the 2D agent; in the other case, Figure 5b describes the 3D agent’s movements.

**Figure 6:**
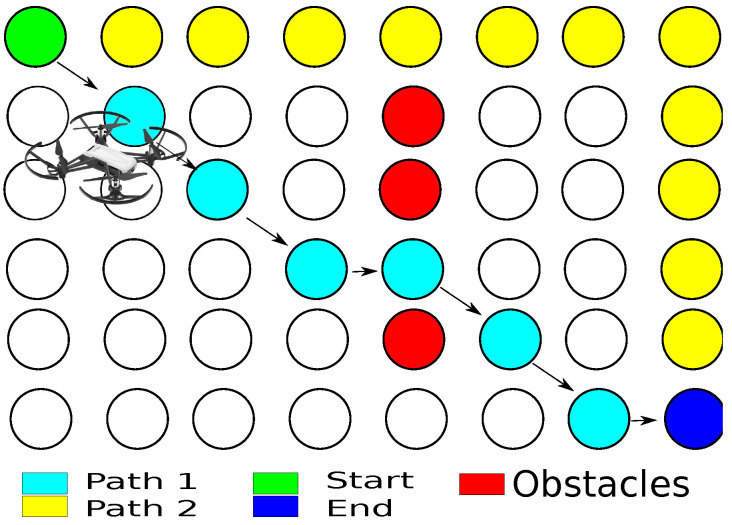
Path planning description using $A^{*}$ algorithm.

**Figure 7:**
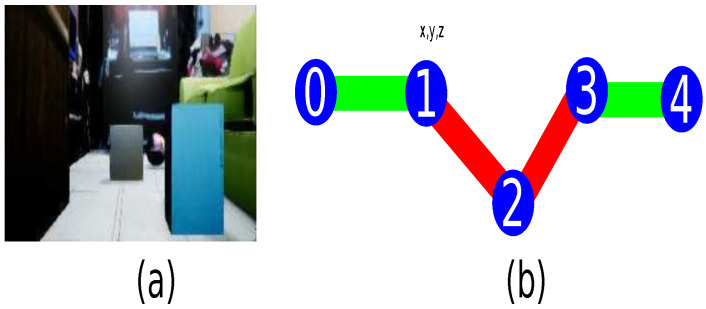
Associated path for each sample. (**a**) Virtual sample. (**b**) Virtual path.

The following system inspired by the image caption problem is proposed, in which a sequence of words describe an image [48]. However, a path generated by the $A^{*}$ algorithm (Figure 6) is associated with a sample at an instant time, as it is shown in Figure 7.

Nevertheless, the $A^{*}$ algorithm has inadequate performance when the number of nodes increases. Therefore, the navigation meshes are used. This approach is a virtual environment-level, mainly employed in video games. A map in a virtual world is composed of triangles, and each triangle forms primitive geometric. Consequently, the map is divided into primitive-shaped meshes [49], reducing the number of nodes based on the centroid of each figure.

### 3.3. Metadata Information for Each Node

Metadata are structured such that they provide context for information on objects of all kinds, including research data, and in doing so, they enable the use, preservation, and reuse of those objects [50]. Hence, the metadata are used to supply the context about position and colors to virtual elements.

As the $A^{*}$ uses graphs constituted by a tuple containing a weight defined from the separation between the nodes. Therefore, it is possible to add more information for each node. Consequently, metadata describes the agent and the moving of each element in the environment. The supplementary information for each node is the distance and the angle for each moving obstacle with the agent.

Likewise, the system determines the estimated time required by the agent to move between the generated sequence with this data and the description using colors to indicate the speed that the agent takes during its flight. Thus, two speeds are: low speed is the minimum flight speed, and the high speed is twice the minimum speed described in the Algorithm 1.

In this way, the time for the entire path is determined as (10) and the time for the path generated by 3D navigation meshes is described in (11). In both cases, the distance between nodes is 20 cm, and the velocity is constant. The Algorithm 2 describes the sequence of actions for generating the dataset.

**Algorithm 1** Algorithm to describe path planning’s features.**Input:** set of 500 virtual samples. **Output:** path’s description with virtual elements.  *Initialization*:
1:Load dataset.*Loop training*2:**for**i=0 to N−1 **do**3:    save agent’s position and angles with respect to obstacle4:    save position of obstacles in motion5:    **if** $Node_{i}$ is centroid and $Node_{i+1}$ is centroid **then**6:        paint the line with blue color7:        velocity = two times constant velocity8:    **else**9:        paint the line with red color10:        velocity = constant velocity11:    **end if**12:**end for**13:**return** list of nodes with describe the path


**Algorithm 2** Algorithm for generating dataset.**Input:** Define the behavior of each obstacles in the environment. **Output:** set of estimated path.  *Initialization*:
1:Multi rotor executed on original position.*Loop generating path*2:**for**i=0 to *N* **do**3:    Set the position of virtual multi rotor and take a sample.4:    Estimate the position of each obstacles.5:    Define the occupied spaces.6:    Run $A^{*}$ algorithm full.7:    **if** Exist a interception between ($N_{i}$ and $N_{i+1}$) **then**8:        Run path with mesh navigation origin position $N_{i}$ to destination $N_{i+1}$.9:    **end if**10:**end for**11:**return** Set of image with path generated by $A^{*}$.

**Definition** **4.** *Let a number of nodes N and a constant velocity velocity. The time in full path is the sum of the difference between the position of the current node and the next node divided by a constant velocity.*

(10) $$time_{full}=\sum_{i=0}^{N-1}\frac{NodePosition_{i}-NodePosition_{i+i}}{velocity}$$

**Definition** **5.** *Let a number of nodes near to object in motion N, a number of 3D mesh navigation centroid M, and a constant velocity velocity. The time with 3D mesh navigation path is the sum of the difference between the position of the current node and the next node divided by a constant velocity, and the sum of the difference between the position of the current centroid with the next centroid divided by two-times a constant velocity.*

(11) $$time_{meshNav}=\sum_{i=0}^{N-1}\frac{NodePosition_{i}-NodePosition_{i+1}}{velocity}+\sum_{m=0}^{M-1}\frac{NodePosition_{m}-NodePosition_{m+1}}{2velocity}$$

### 3.4. End-to-End Approach Using an Auto-Encoder

**Figure 8:**
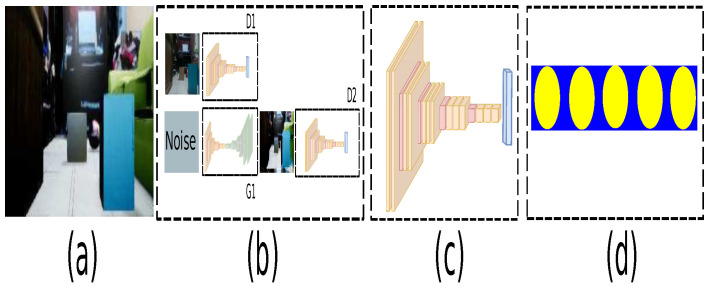
Domain change system. (**a**) Input sample. (**b**) Domain change by a GAN. (**c**) Features extraction network by deep convolutional network. (**d**) Generated path network by recurrent network.

**Figure 9:**
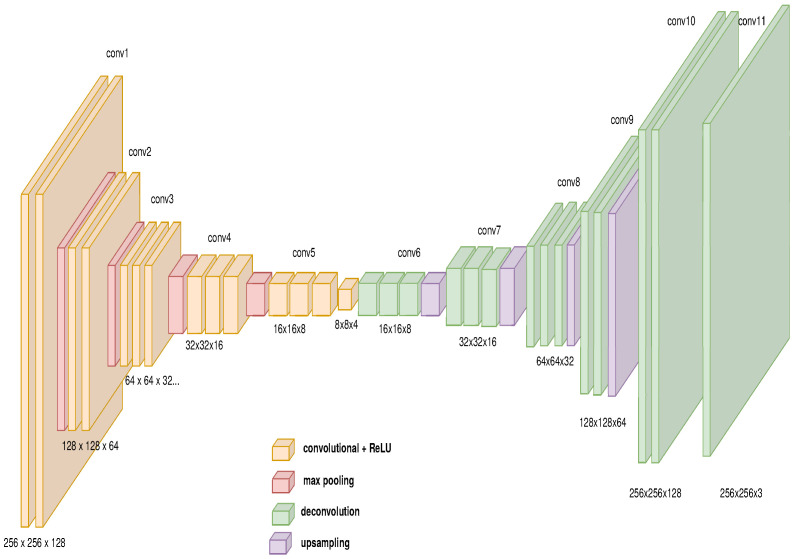
Autoencoder model’s features for GAN’s generator.

**Figure 10:**
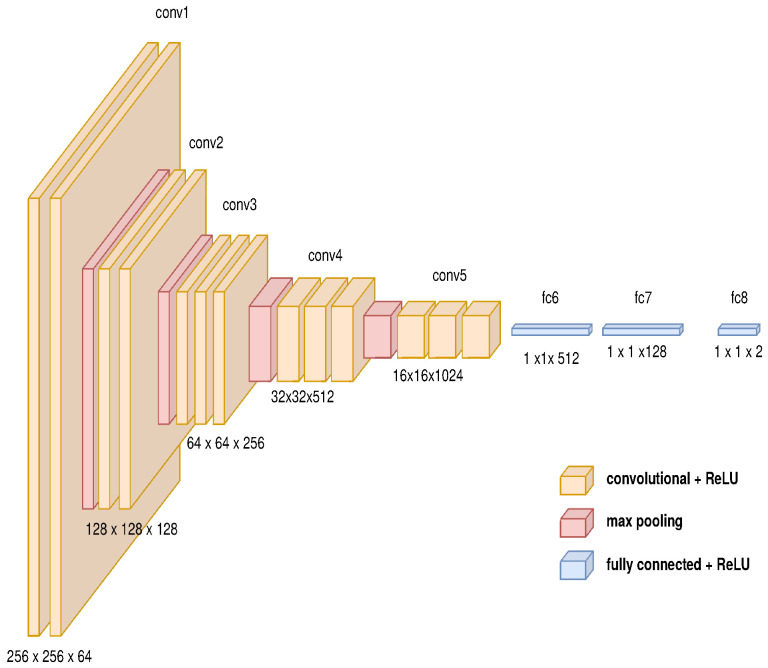
Convolutional model’s features for GAN’s discriminator.

The architecture for the path generator is composed of three main modules as described in Figure 8. The first module is a GAN to change the domain from physical to virtual composed by an auto-encoder as the generative network described in Figure 9 and a deep convolutional network as the discriminator network shown in Figure 10 (the diagrams of the deep neural network were based on https://github.com/kennethleungty/Neural-Network-Architecture-Diagrams (accessed on 1 October 2021)). This module uses 500 samples and calculates the cross-entropy error defined in Equation (12).

(12) $$-\sum_{i}^{C}t_{i}\cdot log\left(f{\left(s\right)}_{i}\right)$$

**Figure 11:**
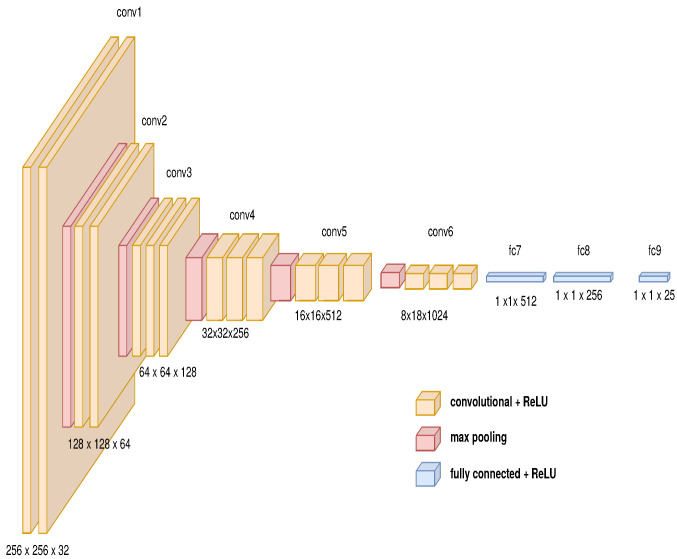
Convolutional model’s features for generating characteristic vector.

**Figure 12:**
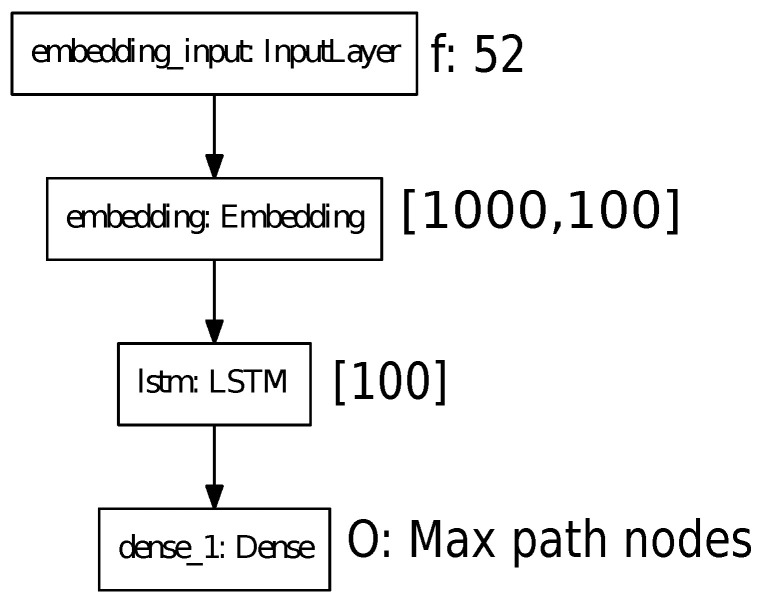
Recurrent neuronal network model’s features for generating path.

The second module determines the characteristic vector using deep convolutional neural networks, as shown in Figure 11. The last module is composed of the architecture of recurrent neuronal network, which is described in Figure 12.

**Figure 13:**
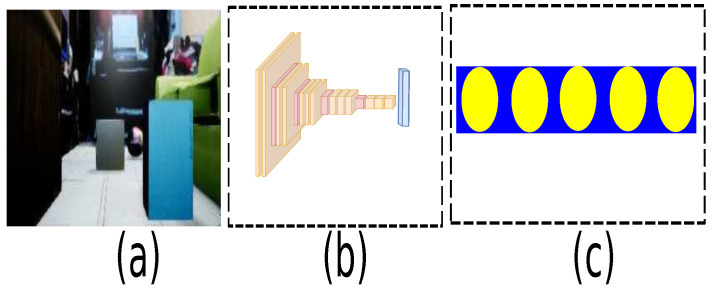
Proposed architecture with transfer learning. (**a**) Input sample. (**b**) Transfer learning network. (**c**) Generated path network.

**Figure 14:**
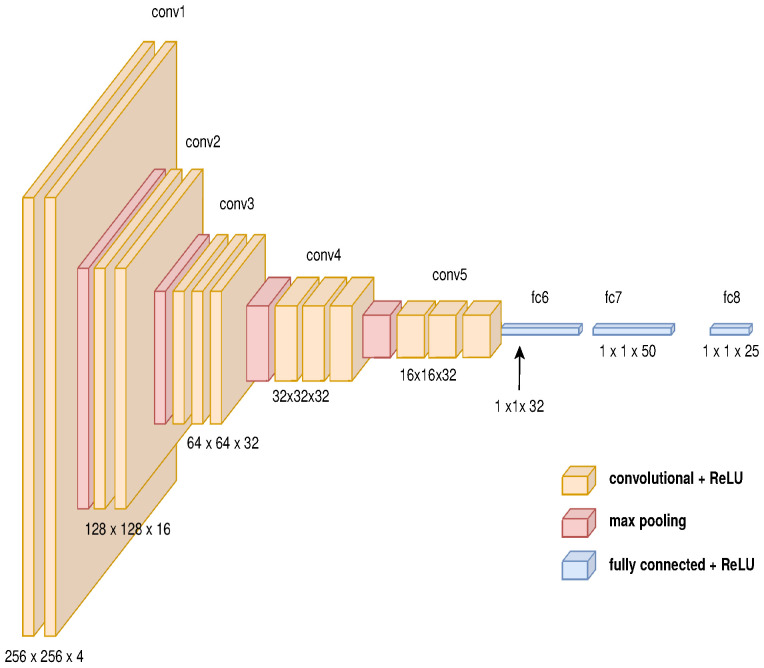
Convolutional model’s features for an embedded devices with transfer learning.

Due to the architecture requiring many operations, the transfer learning approach replaces a high-dimensional model with a model that requires fewer features. In this way, the number of modules is reduced as described in Figure 13. The features of the transfer learning module are defined in Figure 14.

**Figure 15:**
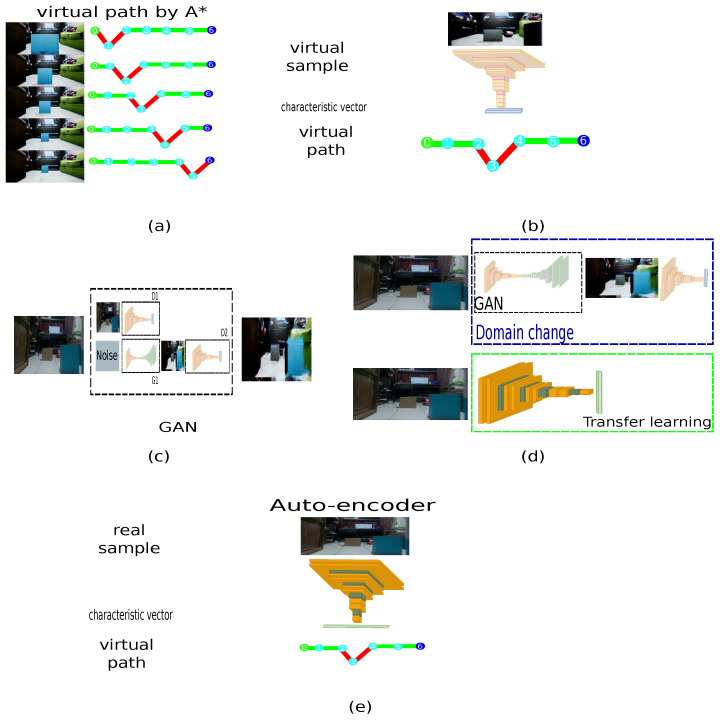
Interoperability between real and virtual environments by a GAN. (**a**) Each virtual sample has an associated virtual path based on $A^{*}$ with metadata. (**b**) An autoencoder estimates the virtual path. (**c**) GAN to connect the real and virtual domains. (**d**) Transfer learning approach to reduce the domain change generated by the GAN. (**e**) The transfer learning model replaces the original to estimate the characteristic vectors, connecting real samples to virtual paths with metadata information.

On the other hand, Figure 15 describes the procedure of the different phases that compose the system. For each virtual sample, a route generated with metadata is associated, as shown in Figure 15a. Subsequently, the proposed auto-encoder is trained to generate a virtual path for each virtual sample, as shown in Figure 15b. Figure 15c illustrates the domain connection using a GAN. Because domain switching and path generation requires a lot of processing time, it is replaced by an architecture with fewer operations based on the *transfer learning* approach, shown in Figure 15d with 50 training samples. Subsequently, Figure 15e describes the change domain from a physical world sample to generate a virtual path with metadata for embedded systems.

## 4. Implementation into a Controlled Real Environment

The following step is to deploy the training in an authentic environment on embedded devices using virtual samples. Therefore, a coefficient aims to determine the minimum number of samples.

### 4.1. Interoperability Coefficient Composed by Image Quality and Join Entropy

**Figure 16:**
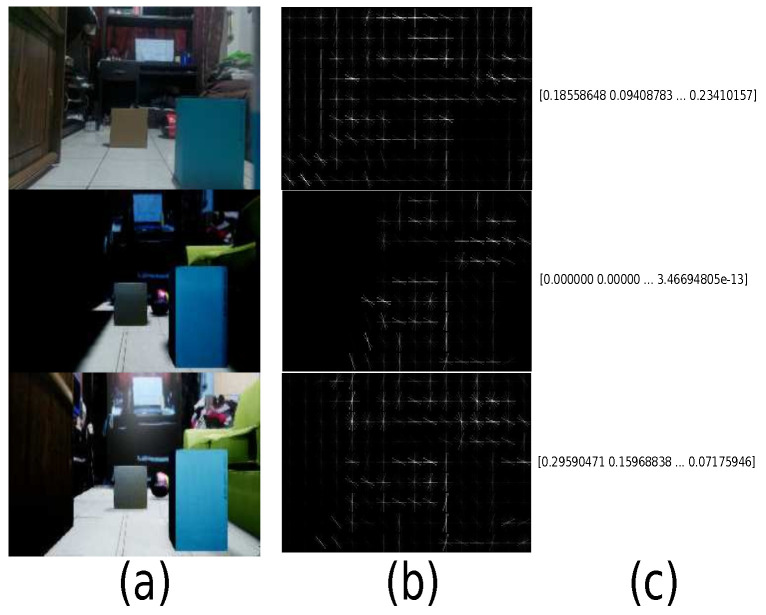
Kind of samples. (**a**) Real sample, (**b**) virtual representation with an essential light source, and (**c**) virtual sample with lights and materials.

One of the tools for assessing the correlation between two images is the HOG [51]. This algorithm allows measuring the comparison of authentic and its virtual representation. This tool obtains a characteristic vector for each of the samples and offers a coefficient that indicates the similarity level, whose hyper-parameters are: orientation equal to 8, pixels per cell equal to 32 × 32, and cells per block equal to 4 × 4. For example, Figure 16 shows a physical sample and its virtual representation with two different detail levels. We observed that the first variation has essential lighting, and the second has a more significant number of directional lighting sources and materials that give more realism to the virtual environment.

Table 1 shows correlation measurements between 30 real-world samples and their virtual representation with two different detail levels. Since lights increase detail level, the correlation coefficient of more detailed samples (lights and materials) is higher than essential light source samples. However, the correlation coefficient between virtual samples created with video game engines and real examples is not high enough. Consequently, representation of the real world is inadequate.

The coefficient described in Equation (13) is composed of the image quality obtained by HOG, and the join entropy generated by the GAN. Table 2 describes that the performance improves when the number of samples increases. Hence, it is determined the number of minimum samples of the authentic environment.

**Definition** **6.** *The interoperability coefficient is composed of HOG determined by the detail of virtual representation multiplied by the entropy of a virtual sample with its generated samples. Let $N_{real}$ be the number of real samples, $x_{r}$ be the real image, and $x_{v}$ be the virtual image for calculating the average HOG relation between both samples. Let $N_{GAN}$ be the number of samples gendered by GAN, y as the real sample’s virtual representation, $y^{\prime }$ like a fake sample, $P{(}_{YY^{\prime }})$ as the probability between a real and fake sample, H(y) as the entropy real sample’s virtual representation, and $H(y^{\prime })$ as the fake sample’s entropy for determining the average join entropy for each step and the sum of the entropy of both samples.*

(13) $$C_{interoperability}=\frac{\sum_{k}^{N_{real}}HOG\left(x_{r_{k}},x_{v_{k}}\right)}{N_{real}}\cdot \left(1-\frac{\sum_{i}^{N_{GAN}}\frac{-\sum_{y_{i}}\sum_{y_{i}^{\prime }}P_{Y_{i}Y_{i}^{\prime }}\left(y_{i},y_{i}^{\prime }\right)logP_{Y_{i}Y_{i}^{\prime }}\left(y_{i}y_{i}^{\prime }\right)}{H\left(y_{i}\right)+H\left(y_{i}^{\prime }\right)}}{N_{GAN}}\right)$$

For this case, the HOG correlation is 0.5490, and the interoperability coefficient is 0.50030 in 58 real-world samples. For this reason, it is recommended to take the number of samples when the interoperability coefficient is upper than 0.50. Thus, the details in the virtual representation are lower than the authentic sample. The virtual representation must have enough information that allows deep learning [52] to use textures. Furthermore, according to the results, if the number of virtual representation samples increases their details in light and material, the interoperability coefficient must increase, and the number of samples can be less. When the joint entropy is low, the data dispersion is similar between the GAN architecture and virtual representation samples.

**Figure 17:**
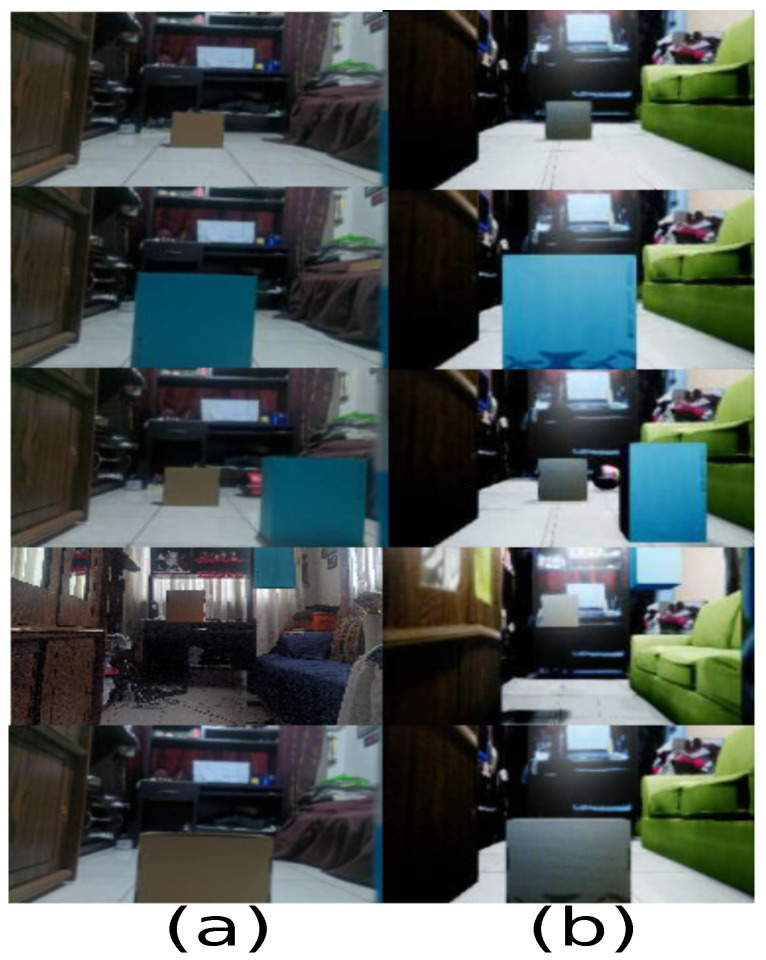
Trained samples. (**a**) Real samples; (**b**) virtual representations.

For example, Figure 17 describes the samples. Therefore, some real samples are displayed with their respective virtual representation, where real samples are captured based on the logic of the scenario, but there may also be exceptional or unlikely cases that in the real world cannot happen or are very difficult to occur. In this experiment, boxes are used to represent obstacles on the ground. Even the obstacles can get rare cases as being in the air and being considered by the simulator.

### 4.2. Virtual Dataset

The following step is to associate each sample with a path generated by the path planning algorithm. In addition, the agent used to move in the scenario is a *DJI Tello* drone whose minimum displacement is 20 cm. For this reason, the discrete algorithm $A^{*}$ was selected.

**Figure 18:**
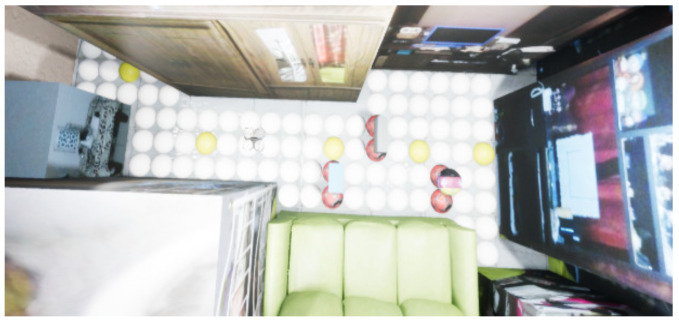
Representation of movements in 2D plane for discrete algorithm $A^{*}$.

The trajectory generator constructs a path in a 3D scenario. Hence, considering the characteristics of the $A^{*}$ algorithm, the jumps used are 20 cm. Figure 18 shows the distribution of the 92 points where the agent can move in a 2D plane.

**Figure 19:**
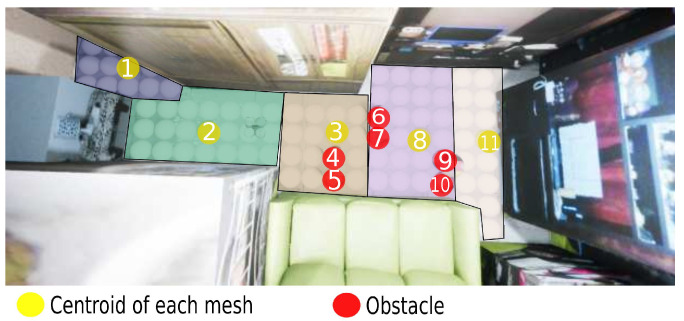
Meshes navigation in a 2D plane.

In this way, an adequate performance is kept using the $A^{*}$ algorithm because one of its characteristics is that the performance decreases when the number of nodes increases. In this case, Figure 19 shows that 11 nodes are used instead of 92 nodes in the 2D plan, where the yellow nodes are the centroids, and the red nodes are the object position in motion.

**Figure 20:**
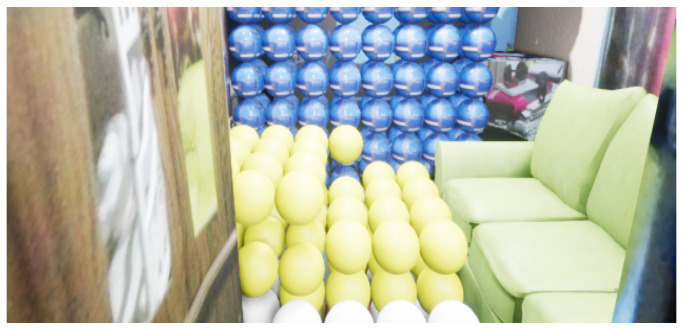
Representation of mesh navigations in 3D space.

Since the problem is a 3D space, the number of movements increases. Consequently, the $A^{*}$ algorithm has difficulties when the number of nodes increases. For this experiment, the total number of nodes is 920, using 3D navigation meshes for grouping spheres belonging to a region into a zone formed by primitive geometry shapes. For example, Figure 20 shows the grouping of 3D navigation meshes for different regions associated with each color.

**Figure 21:**
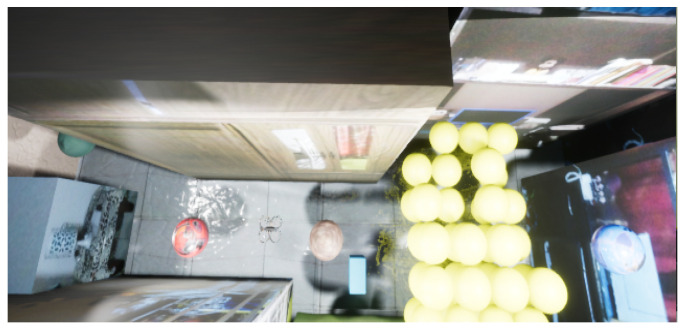
Representation of centroid created by 3D mesh navigations.

Likewise, Figure 21 shows the reduction of the displacement nodes. When moving obstacles generate a possible collision, the 3D navigation mesh can be kept entirely or take a region defined by a radius around the moving object.

Since different issues appeared when the system was implemented on real MAV, a state machine was designed. The MAV transmits and receives the camera samples and the commands to control the vehicle. For example, the MAV requires waiting until the response is received. Therefore, these kinds of issues affect the real-time execution of the system.

**Figure 22:**
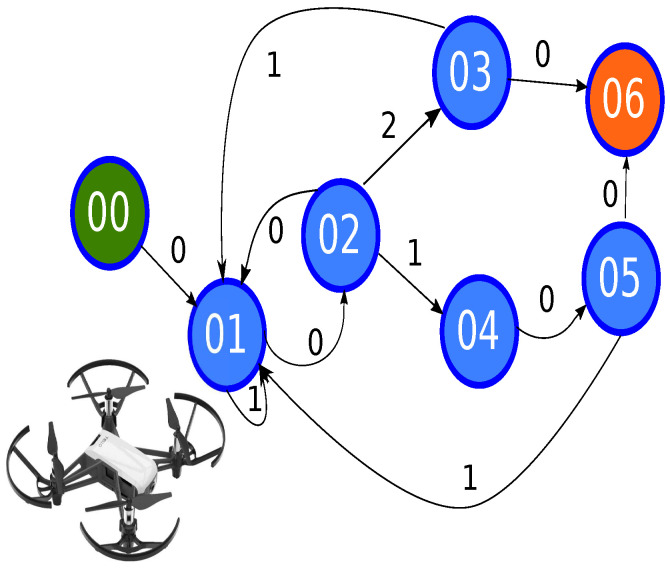
States to control *MAV*.

Due to the starting position of the MAV being at one meter, we defined the first step as placing the drone at a distance of 30 cm ground. Subsequently, a filter was added to avoid external problems generated mainly by the illumination. The filter consists of two stages. The first is to read five samples and evaluate the mean of the following free node since illumination changes add behaviors that disturb the system’s performance. The second step of the filter is that in case of strange behavior such as the generation of five different samples, it must return to its previous state to protect the MAV. In this way, Figure 22 illustrates the state machine, and the Table 3 describes each state and its transition, respectively. Consequently, the diagram facilitates the understanding of the system operation, and the state diagram helps protect the vehicle’s physical state because it avoids abrupt collisions.

## 5. Experimental Results and Analysis

This section describes the performance of the design. The metrics are selected based on state-of-the-art. Furthermore, the model’s behavior is shown with an AR approach to observe the generated path with a first-person perspective. On the other hand, an implementation on a real MAV is performed to compare both behaviors.

### 5.1. Path Planning Generator with Metadata Performance

**Figure 23:**
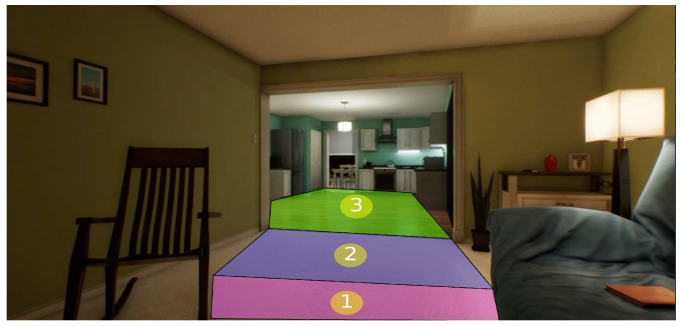
Environment division using navigation meshes for rooms 1 and 2.

**Figure 24:**
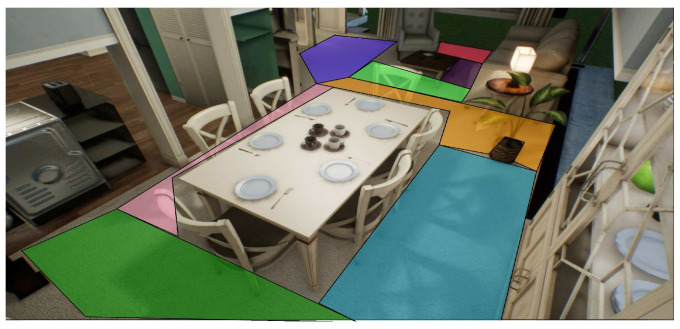
Environment division using navigation meshes for room 3.

To describe the behavior of this experiment, the following 3D sources were used (the 3D environment was taken in the free section of the unreal market with the keyword house https://www.unrealengine.com/marketplace/en-US/store (accessed on 3 December 2020)). The scenario is divided into navigation meshes to reduce nodes for each path, as it is shown in the Figure 23 and Figure 24. Therefore, the performance is compared with the algorithm Q-learning [53]. We modified the framework AirSim to operate a MAV with a size of 20 cm.

According to [54], one principal feature for measuring path planning is the number of collisions. Due to the auto-encoder generating a sequence of nodes corresponding to a vector, we suggest metrics to compare vectors between a expected vector *x* with an generated vector *y*, such as euclidean distance (14), Manhattan distance, (15) and cosine similarity distance (16). Besides, a free path coefficient measures whether an obstacle appears on the path defined by (17).

(14) $$euclidean=\sqrt{\sum_{i=0}^{k}{\left(\vec{x_{i}}-\vec{y_{i}}\right)}^{2}}$$

(15) $$manhattan=\sum_{i=0}^{k}\left|(\vec{x_{i}}-\vec{y_{i}})\right|$$

(16) $$cosine\;similarity=\frac{\vec{x}\cdot \vec{y}}{∥\vec{x}∥∥\vec{y}∥}$$

(17) $$C_{free\;collision}=1-\frac{\sum_{i=1}^{N_{samples}}\left\{\begin{array}{c} if\;exists\;collision\;c=1\\ else\;c=0 \end{array}\right.}{N_{samples}}$$

Although the Q-learning algorithm and the proposed architecture generate paths, both are two different perspectives. For this reason, the system just is compared by the generated paths as vectors. The Q-learning algorithm has taken the midpoint in straight lines to have a better criterion than the expected vector, as is described in Table 4.

It is observed that the Q-learning algorithm performs better since one of its features is deep exploration compared to that of the proposed algorithm. Due to the deep exploration feature, the Q-learning algorithm requires more training time because the proposed architecture uses a conventional algorithm to reduce the analysis time. Besides, the Q-learning algorithm has adequate performance in unknown scenarios, and the proposed solution is known scenarios. Therefore, Table 5 summarizes the main features of the two approaches.

### 5.2. Interoperability Performance

Evaluation criteria are based on error and accuracy metrics proposed by Eigen et al. [55] to evaluate and compare the performance of two domains methods. These metrics are formulated as follows, where $Y_{p}$ is a pixel in-first-domain image *Y* (in-second-domain), ${\hat{Y}}_{p}$ is a pixel in the estimated domain image $\hat{Y}$, and *k* is the total number of pixels for each sample.

**Definition** **7.** *Relative error (rel):*

(18) $$\mathrm{rel}\left(Y_{p},{\hat{Y}}_{p}\right)=\frac{1}{k}\sum_{p=1}^{k}\frac{\left|Y_{p}-{\hat{Y}}_{p}\right|}{Y_{p}}$$

**Definition** **8.** *Average ($log_{10}$) error:*

(19) $$log_{10}\mathrm{error}\left(Y_{p},{\hat{Y}}_{p}\right)=\frac{1}{k}\sum_{p=1}^{k}\left|log_{10}\left(Y_{p}\right)-log_{10}\left({\hat{Y}}_{p}\right)\right|$$

**Definition** **9.** *Root mean-squared error (RMSE):*

(20) $$\mathrm{RMSE}\left(Y_{p},{\hat{Y}}_{p}\right)=\sqrt{\frac{1}{k}\sum_{p=1}^{k}{\left(Y_{p}-{\hat{Y}}_{p}\right)}^{2}}$$

**Definition** **10.** *Accuracy with threshold (t): Percentage of $Y_{p}$ s.t. max($\frac{Y_{p}}{{\hat{Y}}_{p}},\frac{{\hat{Y}}_{p}}{Y_{p}}$)= δ<t, $t\in \left[1.25,1.25^{2},1.25^{3}\right]$.*

Since neural networks are empirical, it is impossible to establish an ideal parameter to determine the correct behavior. The Table 6 shows the performance of the GAN on the six metrics, respectively. This information helps to describe this particular problem from the number of samples limited by the interoperability coefficient. Even though the entropy is low, these metrics measure pixel per pixel position. Therefore, the error is higher than entropy.

**Figure 25:**
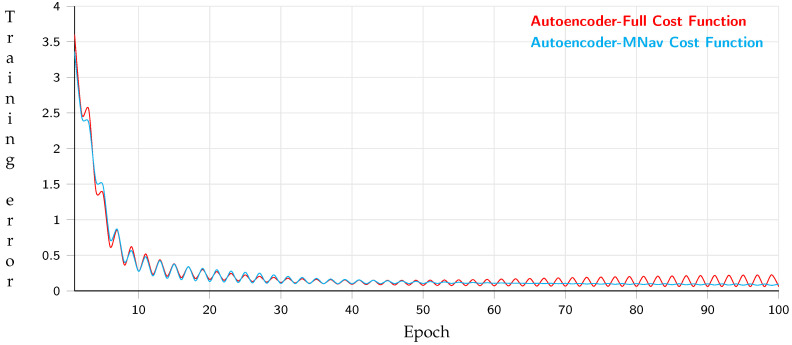
Training behavior of auto-encoder approach using navigation meshes and full path.

Alternatively, Figure 25 describes the training of the architecture used to generate the path. The training is composed of two evaluations. The first one is from the generation of all the nodes proposed by the $A^{*}$ algorithm whose max length is 25 nodes, while the second evaluation uses 3D navigation meshes whose max length is 10 nodes, having 95% precision with 20 test samples. Each node contains the spatial position saved in a dictionary; therefore, each node’s information is reduced. Further, the training has a size batch of 64 samples, evaluating 6.400 times.

Table 7 shows the metric results for the full path and the path with 3D navigation meshes, following the same equation to compare vectors mentioned above. According to the results, the training with navigation meshes converges faster and has the best performance because the number of nodes is lower than the full navigation.

**Figure 26:**
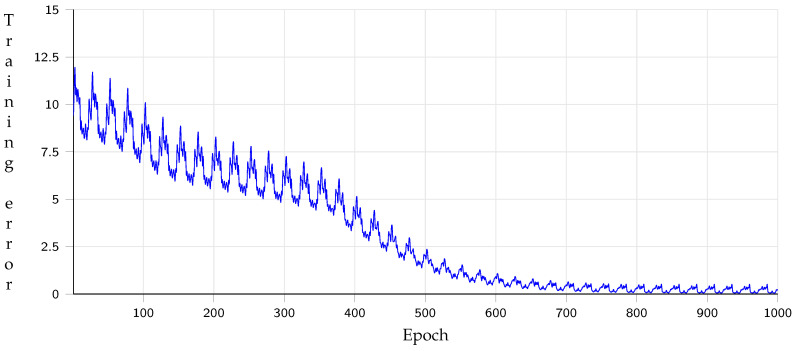
Training behavior of transfer learning approach.

Although the system is acceptable in a desktop computer, the performance drops for an embedded system with limited resources. For this reason, transfer learning is employed. Figure 26 describes the optimization behavior of the error function of the transfer learning model, having 90% of precision with 20 test samples. Besides, the Table 7 describes the evaluation of the path generation by each of the vector comparison metrics with the transfer learning approach in 1000 evaluations. AED-Mnav-TL presents the best performance because the time increases, and the path comprises 3D navigation meshes. Hence, a short path and high performance are obtained.

Due to transfer learning reducing the number of operations, the system is evaluated in different technologies to determine the frames per second. According to [56], when a system succeeds with at least 10 frames per second, it is considered in real-time. Consequently, Table 8 shows the number of frames in different technology in two embedded systems to run models built by Tensor Flow lite. Afterward, the system gets more frames to be considered a real-time system.

### 5.3. Performance through an Augmented Reality System and a Real MAV

**Figure 27:**
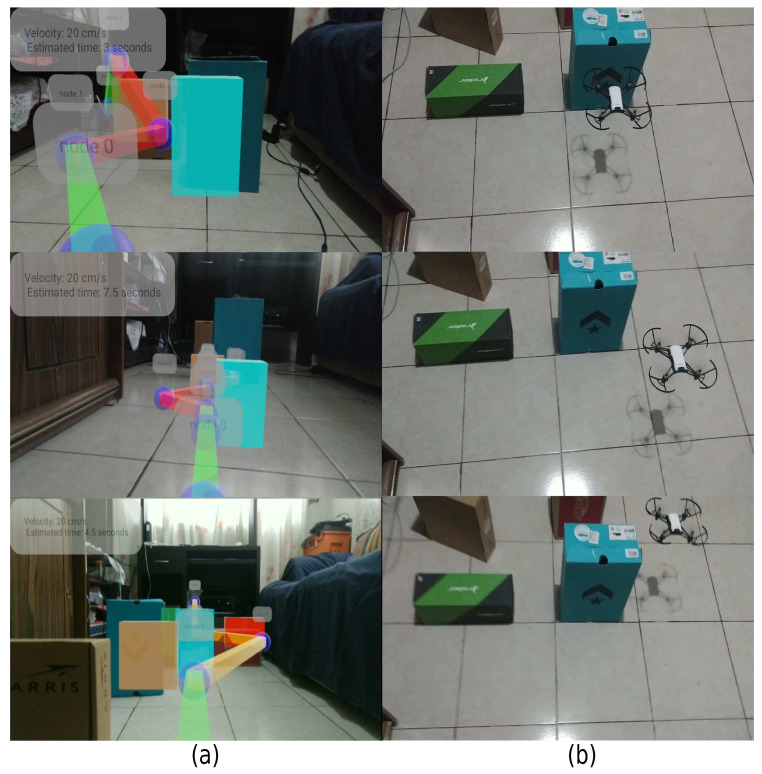
Implemented environments. (**a**) Estimated path through AR. (**b**) Real MAV in motion.

An AR system is designed to describe the interaction with an authentic environment. Figure 27 shows the augmented reality experience using metadata to describe the path’s characteristics. Figure 27a shows the physical sample, and Figure 27b describes the augmented reality experience, as the green color indicates a higher speed than the red color speed. Furthermore, the system represents the objects in motion as virtual boxes, showing the location along the path, and time can be estimated based on node type, as connecting centroid type nodes increases speed.

Subsequently, the solution is employed in two embedded systems and evaluated the free coefficient on 30 samples. Table 9 shows the results obtained. Due to the characteristics of the illumination, the leftovers generated and elements not foreseen in training, originating a decrease in the free coefficient for the AR system in comparison with the training evaluation but maintained a constant *FPS*. Contrarily, the system implemented in a MAV result is lower than AR. Additional factors are observed, such as the field of view of MAV, camera position, and the noise generated by sending the sample for processing.

It is not easy to generalize a model that fits any scenario because neural networks are empirical. Therefore, the number of minimum images varies according to the number of combinations in the scenario. In addition, the rotation for each object may cause the number of minimum samples to increase. For this reason, it is suggested to add possible scenarios that may exist but are unlikely to determine greater diversity in the samples. Besides, the proposed index depends on the rendering quality.

The use of simulators allows obtaining a large amount of data and creates unlikely cases. Likewise, an end-to-end approach allows reducing complex architectures in terms of development time, elaboration, and investment by using data through simulators.

Another factor is the lighting because the shadows generate noise so that the artificial lighting compensates the illumination for the system to perform correctly. One of the situations that affect the performance of the system implementation in a real MAV is the camera transmission sent by the vehicle to the embedded. By the fact of reaching a rate of 30 FPS, the MAV compresses the camera sample to transmit at that speed. Moreover, the samples have a low resolution compared to that of the samples used to construct the virtual representation, and the camera has no autofocus.

The MAV used is not intended for indoor use because its primary function is to take photographs. When capturing photographs, the samples are better than when transmitting video since processing for transfer requires a longer run time than taking a single sample. Although the camera has an angle of −45 degrees and the experiment had successful outcomes, the samples should be of better quality in MAVs that perform processing without transmission. Finally, despite the limited resources of the embedded devices, the functionality was improved and enriched.

This development describes an alternative to reduce time through *GAN* that allows reducing implementation times connecting the training simulation to an authentic environment and reducing costs of development time.

## 6. Conclusions

In this work, we attempted to connect two domains: the authentic world and its virtual representation based on the characteristics of GANs. One of the main problems is the number of samples for each domain. The interoperability coefficient determines the number of samples related to the details and the entropy generated by the GAN. In this way, to avoid performing a virtual representation for each real-world sample.

The metadata was useful to describe the estimated path and position of each obstacle. For example, the AR experience offers in graphical form the behavior of the path from the current agent’s position to the final point because the path was designed to regard the paths generated by the simulator. The experiment proved an adequate performance to interact with a 3D environment considered a real-time system using the end-to-end approach that reduces external elements and the transfer learning approach to minimize the number of operations.

On the other hand, to examine the behavior of this development, the security level-based path planning problem was used to avoid collisions. The collision-free coefficient was evaluated to measure the safety level performance. According to the results, the system performed adequately. However, factors such as image transfer to a server, illumination, and shadows restrict this solution’s scope because they directly impact the samples. Therefore, it is recommended to have constant sources of illumination.

This development aims to propose complex solutions from a reduced source of information from known scenarios enriched with metadata information during the training. However, it is necessary to carry out more tests in a more significant number of conditions to increase the level of security and to have a greater scope.

As future work, it is necessary to analyze the samples with lighting variation to have more variation and reduce the error generated by the variations of external light sources. Furthermore, it is crucial to increase the number of conditions and design more scenarios to evaluate the performance with greater diversity in the samples. 

## Figures and Tables

**Table 1:** Correlation between real-world and virtual samples.

	Simple Environment Virtual-Real	Environment with Lights-Materials Virtual-Real
Factor Correlation (mean)	0.3708	0.5490
Factor Correlation (std)	0.0824	0.0755

**Table 2:** Minimum real samples estimation for building VR environment.

Number of Samples	Join Entropy	Interoperability Coefficient
10	0.8956	0.05731
20	0.6071	0.21570
30	0.3075	0.38018
40	0.2197	0.42384
50	0.1302	0.47752
58	0.0887	0.50030

**Table 3:** Description of states diagram for flying a MAV.

State	Transition	Description
00	$\left\{0\right\}$	Set the MAV MAV 30 cm above the surface
01	$\left\{0,1\right\}$	Transition 0: get five samples and taking the mean of following movement Transition 1: the response time failed, reset counter
02	$\left\{0,1,2\right\}$	Transition 0: return to previous state, the movement is randomized Transition 1: diagonal movement Transition 2: the straight movement
03	$\left\{0,1\right\}$	Transition 0: go to final state Transition 1: movement to following node
04	$\left\{0\right\}$	Complete the diagonal movement
05	$\left\{0,1\right\}$	Transition 0: go to final state Transition 1: movement to following node
06	-	Final state

**Table 4:** Comparative of performance about transfer learning approach and its standard deviation in 50 samples and Q-learning algorithm. Where ↓ Low is better, and ↑ Up is better. AED-MNav-TL (auto-encoder with 3D mesh navigation—transfer learning).

Model	Accuracy Euclidean Distance (Mean-Std) ↓	Accuracy Manhatan Distance (Mean-Std) ↓	Accuracy Cosine Similarity (Mean-Std) ↓	Coefficient Free Collision ↑
AED-MNav-TL	1.7485 ± 0.2856	4.3214 ± 2.1967	0.2145 ± 0.0473	0.92
Q-learning	1.5841 ± 0.3658	4.8415 ± 1.9927	0.1847 ± 0.0308	0.96

**Table 5:** Comparative of features about auto-encoder with Q-learning algorithm.

Feature	Q-Learning	Img2path
Principal issue	Optimize a policy	Associate a conventional algorithm
Training time	Long because of a deep exploration	Short because of a limited exploration
Type of environment	Unexplored environments	Known environments
Size path	Long because of limited movements	Short because of navigation meshes

**Table 6:** Performance about quantitative metrics in GAN and its standard deviation in 50 samples. Where ↓ Low is better, and ↑ Up is better.

rel—std ↓	rms–std ↓	$\log_{10}$–std↓	$\delta_{1}$–std↑	$\delta_{2}$–std↑	$\delta_{3}$–std↑
0.8981–0.8452	0.9141–0.4889	0.4331–0.1245	0.6668–0.0202	0.8219–0.03458	0.8719–0.0318

**Table 7:** Performance about transfer learning approach and its standard deviation in 50 samples. Where ↓ Low is better, and ↑ Up is better. AED-Full (auto-encoder with full path), AED-MNav (auto-encoder with 3D mesh navigation), AED-full-TL (auto-encoder with full path—transfer learning) and AED-MNav-TL (auto-encoder with 3D mesh navigation—transfer learning).

Model	Accuracy Euclidean Distance (Mean-Std) ↓	Accuracy Manhatan Distance (Mean-Std) ↓	Accuracy Cosine Similarity (Mean-Std) ↓	Coefficient Free Collision ↑
AED-Full	12.1415 ± 1.0488	25.3784 ± 2.2804	0.1573 ± 0.0248	0.88
AED-MNav	2.2649 ± 0.4982	5.9261 ± 1.9159	0.0281 ± 0.0152	0.94
AED-Full-TL	11.7146 ± 1.9251	24.8429 ± 1.5279	0.1414 ± 0.1097	0.86
AED-MNav-TL	1.7267 ± 0.4194	4.7151 ± 1.9014	0.0246 ± 0.0204	0.94

**Table 8:** Frames per second in embedded devices.

Device	Float16 (FPS)
Jetson nano 2G Tensorflow-lite	10
Jetson nano 2G Tensor RT	40
Android device Moto X4 CPU-4 threads	12
Android device Moto X4 GPU	18
Android device Moto X4 NN-API	6

**Table 9:** Free coefficient in real environment.

Augmented Reality Free Coefficient	MAV Free Coefficient
0.7666	0.5666

## Data Availability

Not applicable.
